# 

**Published:** 1911-11

**Authors:** 


For Texas Medical Journal.

### THOUGHTS.

Thoughts, tho' little things
Are yet possessed of wings
That flit now here, now there
As free as sun-kissed air!

How great may be the harm
Or yet the wondrous charm
Unwonted they may do
To me, or yet to you!

Then let us hourly strive
To make our minds a hive
From which these little things
May fly, without their stings.

—Victoria A. H. Duggan.

